# *In silico* and *in vitro* analysis of an *Aspergillus niger* chitin deacetylase to decipher its subsite sugar preferences

**DOI:** 10.1016/j.jbc.2021.101129

**Published:** 2021-09-01

**Authors:** Martin Bonin, Lisanne Hameleers, Lea Hembach, Thomas Roret, Stefan Cord-Landwehr, Gurvan Michel, Bruno M. Moerschbacher

**Affiliations:** 1 University of Münster, Institute for Biology and Biotechnology of Plants, Münster, Germany; 2 Sorbonne Université, CNRS, FR 2424, Station Biologique de Roscoff (SBR), Roscoff, France; 3 Sorbonne Université, CNRS, Integrative Biology of Marine Models (LBI2M), Station Biologique de Roscoff (SBR), Roscoff, France

**Keywords:** chitosan, carbohydrate function, carbohydrate biosynthesis, crystal structure, enzyme mechanism, molecular dynamics, molecular docking, CDA, chitin deacetylase, CE4, carbohydrate esterase 4, ClCDA, *Colletotrichum lindemuthianum* CDA, CnCDA4, *Cryptococcus neoformans* CDA, COS, chitooligosaccharide, DA, degree of acetylation, DP, degree of polymerization, GlcN/D, glucosamine, GlcNAc/A, *N*-acetylglucosamine, GPI, glycosylphosphatidylinositol, HILIC, hydrophilic liquid interaction chromatography, MD, molecular dynamics, PA, pattern of acetylation, paCOS, partially acetylated COS, PDB, Protein Data Bank, RMSF, root mean square fluctuation, VcCDA, *Vibrio cholerae* CDA

## Abstract

Chitin deacetylases (CDAs) are found in many different organisms ranging from marine bacteria to fungi and insects. These enzymes catalyze the removal of acetyl groups from chitinous substrates generating various chitosans, linear copolymers consisting of *N*-acetylglucosamine (GlcNAc) and glucosamine. CDAs influence the degree of acetylation of chitosans as well as their pattern of acetylation, a parameter that was recently shown to influence the physicochemical properties and biological activities of chitosans. The binding site of CDAs typically consists of around four subsites, each accommodating a single sugar unit of the substrate. It has been hypothesized that the subsite preferences for GlcNAc or glucosamine units play a crucial role in the acetylation pattern they generate, but so far, this characteristic was largely ignored and still lacks structural data on the involved residues. Here, we determined the crystal structure of an *Aspergillus niger* CDA. Then, we used molecular dynamics simulations, backed up with a variety of *in vitro* activity assays using different well-defined polymeric and oligomeric substrates, to study this CDA in detail. We found that *Aspergillus niger* CDA strongly prefers a GlcNAc sugar unit at its −1 subsite and shows a weak GlcNAc preference at the other noncatalytic subsites, which was apparent both when deacetylating and *N*-acetylating oligomeric substrates. Overall, our results show that the combination of *in vitro* and *in silico* methods used here enables the detailed analysis of CDAs, including their subsite preferences, which could influence their substrate targets and the characteristics of chitosans produced by these species.

Chitosans are highly versatile and promising biopolymers, consisting of β-1,4 linked *N*-acetylglucosamine (GlcNAc, A) and glucosamine (GlcN, D) units. They are found not only in the cell wall of several pathogenic fungi, possibly masking the fungal chitin to evade the host's immune system (1, 2, 3, 4) but also in other nonpathogenic fungi (5). Furthermore, chitosans can be used in a variety of applications, for example, in agriculture, where they show plant-strengthening and plant-protecting effects, or in the medical field in drug-delivering nanoparticles (6, 7, 8). Their utilization in these areas as well as, presumably, their biological functions highly depend on their physicochemical properties, which are known to be strongly influenced by the percentage of acetylated units (degree of acetylation [DA]) and the length of the polymer (degree of polymerization [DP]) (9, 10). Beyond these two parameters, whose control was the critical step in developing reliably performing second-generation chitosans, the pattern of acetylation (PA) is currently gaining increasing attention (11). A deep influence of the distribution of GlcNAc and GlcN units along the chain, ranging from alternating to random and blockwise, has recently been shown for the physicochemical properties as well as the biological activities of partially acetylated chitosans (12, 13). The DP and DA can be controlled in chemical chitosan production, when either highly acetylated chitin polymers are partially deacetylated, for example, using sodium hydroxide at high temperatures or fully deacetylated polyglucosamines are partially *N*-acetylated, for example, using acetic anhydride (14, 15). However, regarding the PA, only random distributions can be achieved using chemical production methods (16). Therefore, enzymatic production routes using chitin deacetylases (CDAs) have been proposed, which may yield polymers with defined nonrandom PA, and thus, may open the way to third-generation chitosans (12, 17).

According to the carbohydrate-active enzymes database, CDAs (Enzyme Commission: 3.5.1.41) are classified in the carbohydrate esterase 4 (CE4) family, together with four other deacetylase and esterase activities (18). *In vivo*, CDAs catalyze the release of acetate from GlcNAc units of chitins and chitosans (forward mode), but *in vitro*, they are also able to catalyze the reverse reaction, thus *N*-acetylating chitosan polymers and oligomers (reverse mode) (19). They are often found in multigene families suggesting different physiological functions such as the production of cell wall chitosan or the deacetylation of oligomers released from the cell wall (2, 3, 20). All CE4 enzymes are metalloenzymes that share a similar fold and five conserved motifs forming the substrate-binding site. This binding site is comprised of several subsites, with subsite 0 binding the sugar unit being deacetylated and the minus and plus subsites accommodating the units toward the nonreducing and reducing ends, respectively (21). Depending on the number and accessibility of these subsites, the enzymes can act on oligomeric or polymeric substrates and thereby deacetylate either one or several sugar units (22). While it seems that CDAs keep their regioselectivity on chitooligosaccharides (COSs) and partially acetylated COS (paCOS) (23, 24), they appear to generate different patterns on polymeric substrates when acting in forward (17) or reverse mode (12). For the latter, the subsite preferences for an acetylated or deacetylated unit were proposed to play a crucial role (11).

However, subsite preferences have rarely been studied in CDAs. Only a *Colletotrichum lindemuthianum* and a *Cryptococcus neoformans* CDA (ClCDA and CnCDA4) were described to have a clear preference for a GlcNAc unit at subsite −2 or a GlcN unit at subsite −1, respectively (2, 21). So far, CDAs and other CE4 enzymes were mainly tested for their activity on different polymeric substrates like chitins, chitosans, acetyl xylans, peptidoglycans, and their oligomeric counterparts. In recent years, for an increasing number of CE4 enzymes, the mode of action, referring to the different products that are generated over time, was investigated as well (2, 4, 25, 26, 27, 28). Computational methods, such as sequence and structure alignments as well as homology modeling and docking studies, were used to complement these *in vitro* assays (2, 27, 28). To our knowledge, only a few CDAs were in addition studied by molecular dynamics (MD) simulations (22, 29), and only for one CDA, from *Cryptococcus laurentii*, MD simulations were used for a more in-depth analysis (30). The latter, however, was not studied *in vitro*.

In this study, we analyzed AngCDA, an *Aspergillus niger* CDA that is strongly expressed in the mutant *scl-2*, which in contrast to the wildtype forms sclerotia, a survival structure for harsh environmental conditions and a prerequisite for sexual reproduction (31). We solved the 3D structure by X-ray crystallography and analyzed the enzyme using classical *in vitro* assays. In addition, we performed extensive MD simulations, especially focusing on the subsite preferences, which we then validated *in vitro*.

## Results

### Sequence analysis and initial protein characterization

The protein sequence of AngCDA reveals the presence of a CE4 superfamily domain (residues 33–223) including the zinc-binding site and all four catalytic residues previously described for ClCDA (32). Further bioinformatics predictions, including signal peptide, glycosylphosphatidylinositol (GPI) anchor, and transmembrane domains, suggest that the enzyme has a signal peptide (residues 1–19) and is secreted into the extracellular space but is not anchored or attached to the membrane (Figs. S1–S4).

For protein expression in *Escherichia coli* and subsequent purification, two expression constructs without the predicted signal peptide were generated, one with an N-terminal pelB sequence for protein secretion and a C-terminal Strep-tag II for purification and one without a pelB sequence and both an N-terminal and a C-terminal Strep-tag II. The X-ray crystal structure was determined with the first construct, whereas the second construct was used for all activity assays. This latter construct was used as a (laboratory internal) standard construct for better comparability between different CDAs. For the same reason, all experiments were carried out at 37 °C and pH 7. These parameters allowed us to determine the optimum temperature and pH as 50 °C and 8, respectively, using chitotetraose (A4) as a substrate (Fig. S5).

**Figure 1:**
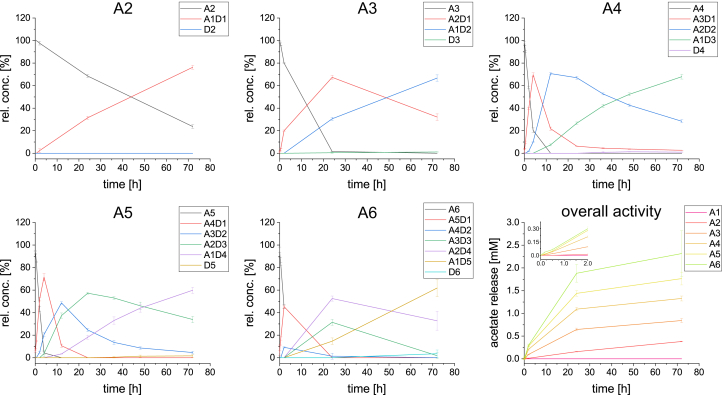
**AngCDA activity on COS.** Product development over time using different COS (0.5 mM) of DP1–6 (A1 not shown) as substrates (n = 3). Graphs show the relative substrate and all product concentrations for a 72-h reaction period determined using MS. The last graph shows the overall activity determined as the acetate release calculated from the MS results, with the inset giving the first 2 h in more detail. COS, chitooligosaccharide; DP, degree of polymerization.

The enzyme activity was first tested on different polymeric and oligomeric substrates, including chitosans with different degrees of acetylation, insoluble α-chitin and β-chitin, colloidal chitin (Fig. S6), and COS of DP1-6 (Fig. 1). AngCDA showed only weak activity on α-chitin and β-chitin, whereas the activity slightly increased on colloidal chitin, probably because of a larger accessible surface area. Overall, the activity on these crystalline substrates was low, as generally seen with other CDAs, too (4, 26, 33, 34). When chitosans with DA12, DA32, and DA46 were used as water-soluble substrates, 62%, 60%, and 48% of the available acetyl groups were removed during 24 h of incubation, respectively. While on chitosan with DA46, the ΔDA increase was linear within the first 4 h, it slowed down toward 24 h, an effect already visible at earlier time points for the other chitosans tested.

When using COS of different DP as a substrate, AngCDA was inactive on monomeric GlcNAc (A1) but active on DP ≥ 2 with an increase toward larger substrates (Fig. 1). For all substrates (A_n_), all intermediate products were produced in succession until only one GlcNAc unit was left (A1D_n-1_). The fully deacetylated product (D_n_) was only produced in very low amounts. Considering that for all tests the same enzyme and substrate concentrations were used, it is striking that the A1D_n-1_ concentration after 72 h always ranged between 60% and 80%, apparently regardless of the number of deacetylations needed until that point. The more detailed time courses for A4 and chitopentaose (A5) reveal that the first deacetylation was the fastest with A_n-1_D1 occurring as the main product after 4 h, whereas A_n-2_D2 was the main product 8 h later for both substrates. This can also be seen when looking at the corresponding peak of the first product (Fig. 1, shown in *red*), which is much sharper compared with the following ones, which get wider with decreasing DA of the corresponding product. The overall activity increased toward larger DPs. However, upon closer inspection of the initial slope of the curves, it increased only up to A5, whereas the initial slope of A6 is similar to that of A5 (Fig. 1; smaller graph in overall activity).

### Crystal structure and multiple structure alignment

**Figure 2:**
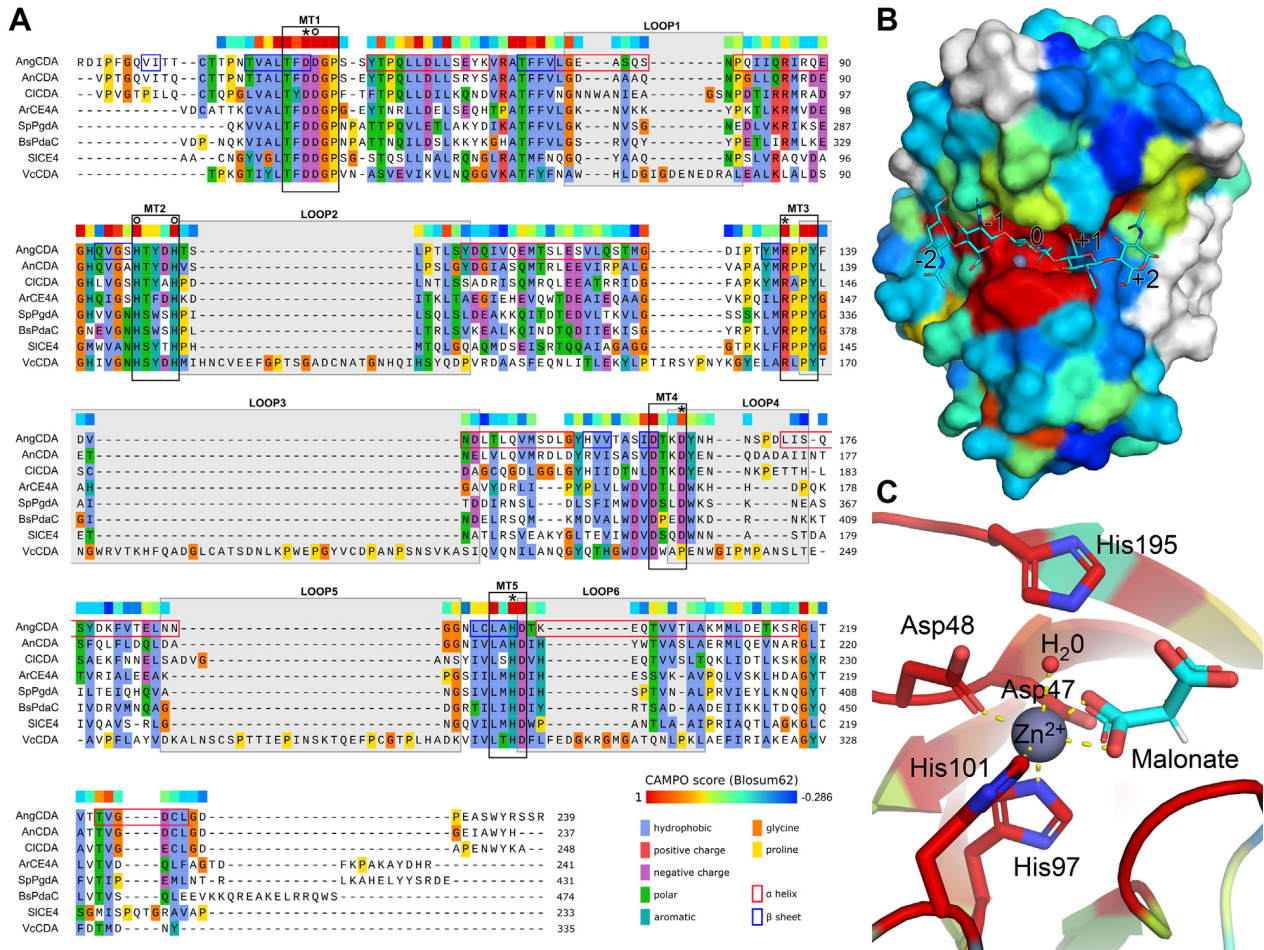
**Sequence and structure comparison to other CE4 enzymes.***A*, structural alignment of CE4 enzymes that are active on chitinous substrates, ranked by their sequence identity to AngCDA. Conserved motifs (MT), containing the metal binding (*circles*) and catalytic (*asterisks*) residues, are highlighted by *black boxes*. The six loops proposed for VcCDA (22) are indicated by a *gray background*. α-helices and β-sheets in AngCDA are indicated by *red* and *blue boxes*, respectively. *B*, AngCDA crystal structure with a chitin pentamer docked *in silico* shown as a surface representation, showing the sugar units −2 to +2. This binding mode corresponds to the first deacetylation of the chitin pentamer *in vitro* as shown in Figure 7*B*. *C*, AngCDA shown as a ribbon diagram, highlighting the zinc ion that is coordinated by the metal-binding triad (Asp48, His97, and His101), a malonate ion, and a water molecule in an octahedral coordination geometry. In addition, the catalytic base (Asp47) and acid (His195) are shown. In both of the structural alignment (*A*) and the 3D representations (*B* and *C*), the residues are colored according to the CAMPO score (47), representing the structural conservation between these enzymes. Further details about the alignment can be found in Multiple structure alignment section. AngCDA, *Aspergillus niger* CDA; CE4, carbohydrate esterase 4; VcCDA, *Vibrio cholerae* CDA.

To further elucidate the substrate-binding site, the X-ray crystal structure of AngCDA was determined at a resolution of 1.81 Å. The enzyme crystallized in the space group *P*432 with one monomer per asymmetric unit. This oligomeric state correlates with the results obtained by gel filtration and dynamic light scattering measurements (28.6 ± 6.5 kDa). AngCDA adopts a distorted (α/β)_8_ fold, as typically found in CE4 enzymes. An intramolecular disulfide bridge (C36–C226), which tethers the N-terminal and C-terminal ends, stabilizes the structure as already observed in the crystal structure of CDAs from the fungal pathogens *C. lindemuthianum* and *Aspergillus nidulans* (Protein Data Bank entries: 2IW0 and 2Y8U) (32, 34). The conserved Asp–His–His triad (Nε2-H97, Nε2-H101, and Oδ1-D48 atoms) plus a malonate ion (O6 and O7 atoms) from the crystallization solution and a water molecule coordinate a zinc(II) ion to form an octahedral coordination geometry with a metal–ligand distance of 2.2 Å. The malonate ion is found at the expected subsite 0, where the acetyl group of the chitinous substrate is normally placed and hydrolyzed (Fig. 2, *B* and *C*).

To compare the structure to other CE4 enzymes, a multiple structure alignment was generated including only those enzymes for which CDA activity has been described in the literature (Fig. 2*A*). All five conserved motifs can be found in AngCDA, with motif 1 harboring the catalytic base (D47) and the metal-binding aspartate (D48) and motif 2 containing both metal-binding histidines (H97 and H101). Motif 3 includes an arginine (R135), which properly orients the catalytic aspartate (D47) and a tyrosine (Y138) forming a hydrogen bond with the acetyl oxygen at subsite 0. Motif 4 contains an aspartate (D165) that enables the protonation of the catalytic histidine (H195), and motif 5 contains a leucine (L193) forming one side of the hydrophobic pocket for the acetamido's methyl group at subsite 0 and the catalytic acid (H195).

All residues mentioned are conserved in all CE4 enzymes included in the alignment, except for the aspartate (D165 in AngCDA), which is a proline in the *Vibrio cholerae* CDA (VcCDA). Both on the sequence and on the structural level, VcCDA differs most from all the other CE4 enzymes (Tables S2 and S3). Its active site is surrounded by six loops, defined by the subsite capping model (22). These loops that are so prominent in VcCDA are very small in the other enzymes, with the exception of a short loop 1 in ClCDA and short loop 4 in AngCDA, AnCDA, and ClCDA (see *gray boxes* in Fig. 2*A*). The subsite capping model suggests that the specific PA generated by VcCDA on oligomeric substrates can be explained by these loops positioning the substrate in a certain way. Of the CE4 enzymes shown in the alignment, solely VcCDA is reported to deacetylate only one sugar unit in the substrate, namely the penultimate unit from the nonreducing end (22). As far as sequence data of the products generated by the other enzymes are available (AnCDA (34), ClCDA (32), ArCE4A (33), BsPdaC (27), and SlCE4 (35)), they are all described to deacetylate several positions in oligomeric substrates. Since for most of these enzymes, different methods and protocols were applied to determine the PA of their products, they are not easily comparable. Nonetheless, their mode of action clearly differs from what is described for VcCDA, since they have a much more open binding site and thus, their mode of action cannot or only partially be explained by the subsite capping model alone. We therefore assume that in addition to the loops, several key residues along the binding site contribute to define the mode of action and regioselectivity of these more open CE4 enzymes.

### Docking studies and MD simulations with A4, A5, and A3D1

**Figure 3:**
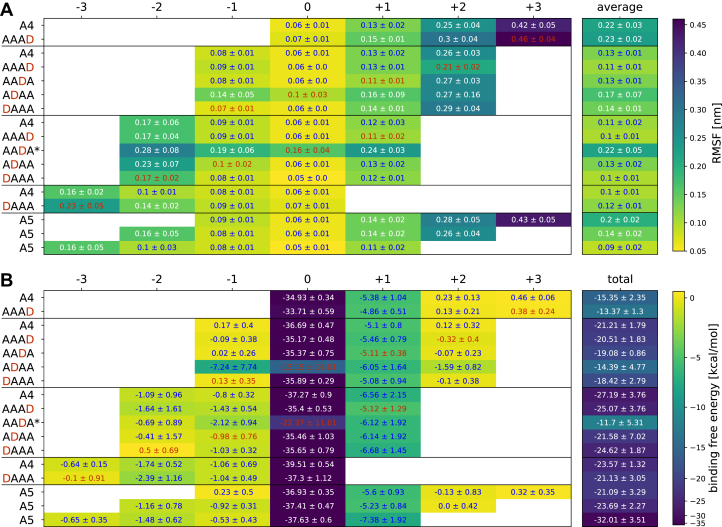
**Substrate fluctuation and binding energy calculated from MD simulations.***A*, root mean square fluctuation (RMSF) of the different substrates and their indicated individual sugar units in the given binding modes. *B*, binding free energy contribution of the different substrates and their indicated individual sugar units in the given binding modes. A more detailed overview of the binding free energy of all active site residues is available in the supporting information 1. The values for GlcN units are highlighted in *red*. All values are means ± SD of nine replicates based on three different starting structures each, except for AADA∗ with n = 6 where the substrate from one starting structure always detached from the enzyme during simulation. MD, molecular dynamics.

To identify amino acids along the AngCDA-binding site, which interact with the substrate, A4, A5, and different monodeacetylated tetramers (A3D1) were docked into the active site of the enzyme. Binding modes spanning from the hypothetical −3 to the hypothetical +3 subsite were chosen, resulting in four different binding modes for A4, sequentially placing each unit at subsite 0, and three different binding modes for A5, placing all internal units at subsite 0. For the A3D1 substrates (DAAA, ADAA, AADA, and AAAD), different binding modes were chosen to create comparable binding modes to A4 (Fig. 3). Since the amino groups of GlcN units are mainly not protonated at pH 7, these substrates were uncharged (36). For each binding mode and substrate, three different conformations with the highest docking scores were chosen from the *in silico* docking. The corresponding docking scores can be found in Table S1.

From hereon, the binding modes will be indicated by the distance of the nonreducing and reducing end sugar units to subsite 0, respectively. For example, an A4 bound with its nonreducing end unit at subsite 0 will be denoted as binding mode [0, +3]. This however does not suggest that a subsite +3 is actually existing. In the following, the term “subsite” refers to the region of the enzyme that interacts with the corresponding sugar unit of the substrate, without implying that these interactions substantially contribute to substrate binding.

The selected complexes from the substrate dockings of all substrates in different binding modes were subjected to MD simulations. As a first indication of the substrate stability in the binding site, the root mean square fluctuation (RMSF) was calculated for each sugar unit and for the complete substrate (Fig. 3*A*). At first, it appears that the fluctuation of each sugar unit solely depends on the subsite at which it is situated and is mostly independent of the substrate length, the binding mode, and even the type of sugar. The sugar unit bound at subsite 0 always shows the lowest fluctuation, whereas fluctuation increases toward both the plus and minus subsites. The average RMSF of the substrate and the fluctuation of the different sugar units suggest that sugar units bound at the minus subsites fluctuated less compared with those at the plus subsites, resulting in an overall more stable binding for binding modes spanning up to subsite −3. On closer inspection, more differences are visible between the different substrates and the different binding modes. The most visible difference is the increased RMSF for paCOS with a GlcN unit at subsite 0, leading to a higher fluctuation of the whole substrate as well. With GlcNAc at subsite 0, the zinc ion coordinates the acetyl oxygen, O3 of GlcNAc, and catalytic water. With GlcN at subsite 0, different coordinations were observed. Either zinc coordinated the amino group and/or the O3 of GlcN, the O6 of the neighboring sugar unit bound at subsite −1, or no part of the substrate. In contrast, a GlcN unit at subsite +1 or +2 seems to slightly decrease the RMSF compared with a GlcNAc unit at the same subsite, whereas this was not the case at subsite +3. The reason for the reduced RMSF might be the missing acetyl group itself, as acetylated amino groups show more rotational movement as long as they do not form a stable interaction with any residue. In all binding modes, the fluctuation of the sugar unit at subsite −2 differs most strongly from that at all other subsites. It appears to be stabilized if another sugar unit is bound at subsite −3 but destabilized if a deacetylated unit is bound at subsite −1. However, a GlcN unit at subsite −2 itself does not seem to influence the fluctuation.

The fluctuation of the substrate serves as a valuable first comparison of the different substrates in their different binding modes. Still, it is not a quantitative measure for the strength of the interaction between substrate and enzyme. Therefore, the binding energy was estimated using the molecular mechanics–generalized Born surface area approach, which calculates not only the total binding free energy but also the contribution of each amino acid and the different sugar units of the substrates. The calculated binding free energy of the sugar units and the total binding free energy for all substrates and binding modes are shown in Figure 3*B*.

**Figure 4:**
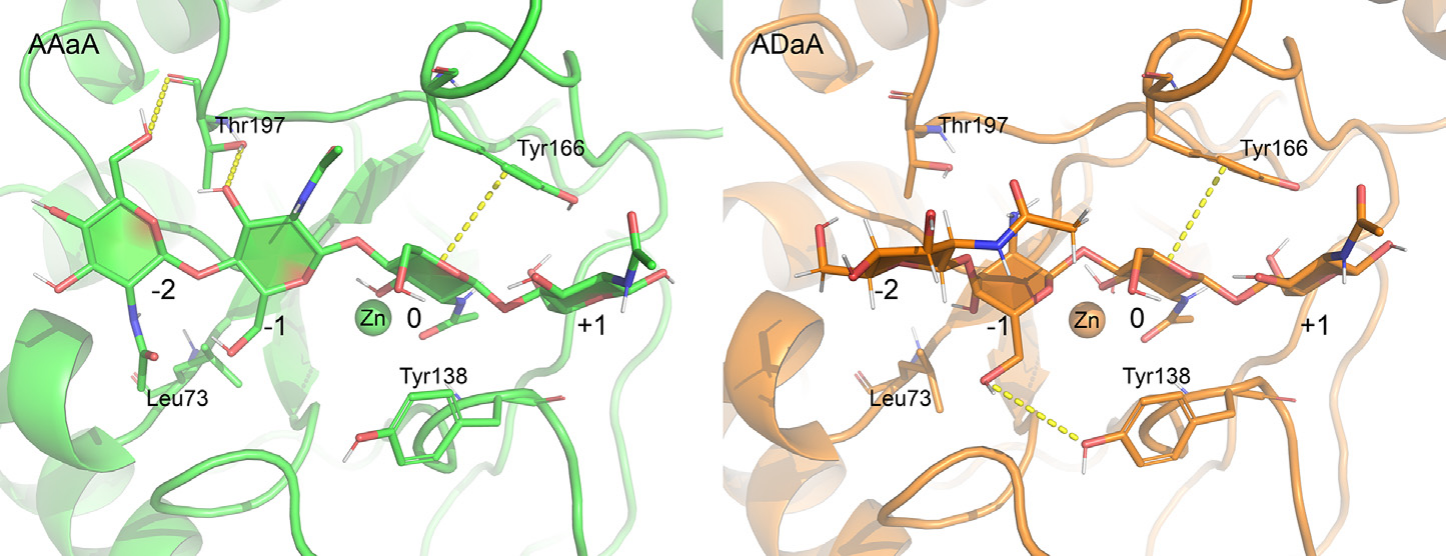
**Comparison between A4 and ADAA bound to AngCDA in the binding mode [−2, +1].** Two representative snapshots are shown, one for A4 (*left*) and one for ADAA (*right*). The AngCDA is shown as a ribbon diagram, with some key residues being highlighted as *sticks* and the zinc ion shown as a *sphere*. *Dashed yellow lines* show interactions with these key residues. The subsites to which the sugar units are bound are indicated next to each unit.

As already indicated by the RMSF results, the calculated binding free energy is the lowest and the binding therefore the strongest, for the subsite 0 sugar unit, and the main differences are not between the different substrates or binding modes but between the different sugar unit positions. Somewhat contradicting the higher RMSF of the sugar units bound toward the plus subsites, the +1 sugar unit strongly contributes to the binding energy. The highest total binding energy, and therefore the most unfavorable binding, was calculated for binding modes [0, +3] and for the binding modes where a GlcN unit is placed at subsite 0. The latter may indicate that deacetylated products may bind in an unproductive way (although a productive binding mode is strongly preferred), leading to product inhibition. The former can be expected to lead to slower rates of deacetylation of the sugar unit at the nonreducing end, as described for the closely related AnCDA (34). Moreover, an acetylated unit appears to be beneficial not only at subsite 0 but also at the other subsites since the total binding free energy increases in all A3D1 substrates compared with A4 with the same binding mode. This effect is most pronounced if a deacetylated unit was placed at subsite −1, which increased the binding energy from −21.21 kcal/mol to −18.42 kcal/mol and from −27.19 kcal/mol to −21.58 kcal/mol for the binding modes [−1, +2] and [−2, +1], respectively. Together with the slightly increased fluctuations of these substrates, this suggests a preference for acetylated units at subsite −1. Interestingly, for ADAA placed with the GlcN unit at subsite −1, the fluctuation and binding energy at this subsite are comparable to A4 in the same binding mode. However, it appears that the GlcN unit at subsite −1 influences the binding of the neighboring sugar unit at subsite −2. Taking a closer look at these simulations, it turns out that the deacetylated sugar unit at subsite −1 is slightly tilted compared with an acetylated unit at the same position, which forces the sugar unit at subsite −2 to fold out of the binding site, allowing a stronger fluctuation (Fig. 4).

**Figure 5:**
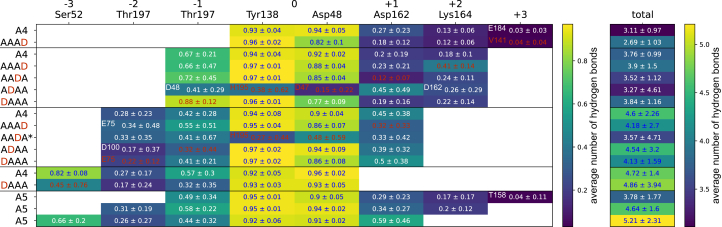
**Analysis of hydrogen bonds derived from MD simulations.** Average number of hydrogen bonds of the indicated amino acids with the sugar units at the corresponding subsites and the total number of hydrogen bonds between substrate and enzyme throughout the simulation for the different substrates and binding modes. Unless otherwise specified in the corresponding field, the value gives the average number of hydrogen bonds between the amino acid named on top and the sugar unit at the indicated subsite. Values for deacetylated GlcN units are highlighted in *red*. All values are means ± SD of nine replicates based on three different starting structures each, except for AADA∗ with n = 6, where the substrate from one starting structure always detached from the enzyme during simulation. A more detailed overview of all hydrogen bonds is available in the supporting information 2. MD, molecular dynamics.

To identify interesting residues that contribute to these observed differences, we took a closer look at the amino acids that showed the strongest influence on the binding energy (see below). In addition, we calculated the average number of hydrogen bonds between substrate and enzyme throughout all simulations and identified the residues with the highest values for each subsite (Fig. 5). It should be noted that a simple cutoff for the hydrogen bond angle between donor, acceptor, and hydrogen atom and for the distance between donor and acceptor was applied. Thus, no direct assumption can be made about the strength of these bonds. The simulations where a GlcN unit is placed at subsite 0, with more movement in the substrate overall, show quite different interactions and are not discussed further. All other simulations show the most stable hydrogen bonds at subsite 0, with the backbone nitrogen of Tyr138 forming a hydrogen bond with the acetyl oxygen. Moreover, the second oxygen from Asp48, the one that does not coordinate the zinc ion, forms a hydrogen bond with O3 of the GlcNAc unit at subsite 0, which is also coordinated by the zinc ion. At subsites −1 and −2, the Thr197 side and main chain oxygens interact with the O3 and O6 of the sugar unit, respectively. This interaction is strongly reduced at subsite −2 if a GlcN unit is present at subsite −1 or −2 since the orientation of these sugar units differs from the others (Fig. 4). Furthermore, a new hydrogen bond between the Tyr138 side-chain oxygen and the O6 of GlcN unit situated at subsite −1 appears, which stabilizes this orientation. At subsite −3, the backbone nitrogen of Ser52 forms a stable hydrogen bond with the acetyl group oxygen. If the acetyl group is missing, only occasional hydrogen bonds with the O4 and O5 were observed. On the plus subsites, mainly Asp162 and Lys164 seem to be involved in hydrogen bond formation with the substrate, whereas no stable hydrogen bonds are formed with the +3 sugar units. It appears that the hydrogen bond with Asp162 is more stable if it interacts with the reducing end sugar, which also leads to a decreased binding free energy at the +1 subsite.

**Figure 6:**
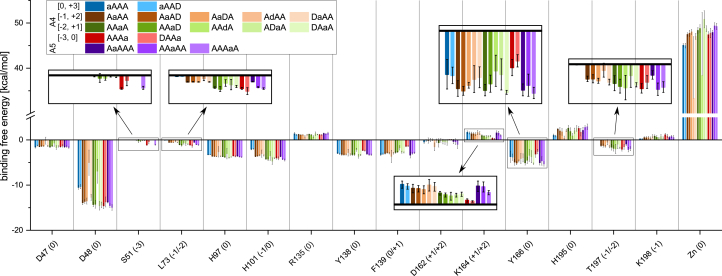
**Influence of most important amino acids for substrate binding.** Average energy contributions of all amino acid residues at the substrate binding site with values below −1 kcal/mol or above +1 kcal/mol, given for all binding modes. The subsite(s) to which each amino acid residue contributes is/are given in parentheses. The different binding modes are grouped by color, with the subsite 0 sugar unit being indicated by a lower case “a” or “d.” Error bars show SD of nine replicates based on three different starting structures each, except for AAdA with n = 6, where the substrate from one starting structure always detached from the enzyme during the simulation. A more detailed overview about all active-site residues is available in the supporting information 3.

All the aforementioned residues belong to those residues that most strongly influence the binding energy (Fig. 6). Besides Asp48 and Tyr138, the catalytic Asp47, the metal binding His97 and His101, the hydrophobic Phe139, and the aromatic Tyr166 contribute to the substrate binding at subsite 0. Tyr166, as studied in detail for the equivalent Trp151 of the cryptococcal CDA by Sarkar *et al.* (30), probably stacks with the substrate. As previously described for ClCDA (32), Phe139 forms a hydrophobic pocket together with Leu193 to accommodate the acetyl methyl group at subsite 0. Leu73 might have a similar role for the acetyl group at the −2 subsite. The positively charged amino acid Arg135 and the catalytic His195 at subsite 0 as well as Lys164 and Lys198 show a positive binding energy because of their high desolvation penalty. This effect, where the exchange of surrounding water molecules by the substrate is energetically disfavored, is by far the strongest for the zinc ion, leading to a strong increase of the binding energy. Upon closer inspection of the values for the different binding modes, it again becomes visible that those with a GlcN unit at subsite 0 stand out. As expected, residues such as Ser51 or Lys164 mostly influence the binding energy in binding modes, where their subsites are occupied. However, it also shows that one residue cannot be simply ascribed to only a single subsite. Lys164 shows not only the strongest influence for binding modes [0, +3], [−1, +2], [−1, +3], and [−2, +2], that is, those involving subsite +2, but also affects, to a weaker extent, binding modes [−2, +1] and [−3, +1], and slightly even [−3, 0], that is, binding modes not involving subsite +2. And even the subsite 0 residues show differences, especially for those binding modes where a terminal unit of the substrate is positioned at subsite 0. Besides the binding modes with a GlcN unit at subsite 0, also ADAA in binding mode [−2, +1] (ADaA, *i.e.*, with a GlcN unit at subsite −1 and an GlcNAc unit at subsite 0, as indicated by the lower case letter a) shows some differences compared with the other [−2, +1] binding modes, especially for Leu73, Tyr166, and Thr197. This seems surprising for Tyr166, as it appears to normally interact with the sugar ring at subsite 0 (Fig. 4). While examining the trajectories with a GlcN unit at subsite −1 more closely, we saw that this tyrosine can rotate toward the minus subsites, occupying the freed-up space of the missing acetyl group.

Overall, many more details can be observed from these simulations, but for space constraints, it is not possible to discuss all of them in detail here. Therefore, all tables generated for the binding free energy calculations and hydrogen bonds are summarized in the two spreadsheets included in the supporting information.

### Mode of action on A4, A5, and D4 studied *in vitro*

**Figure 7:**
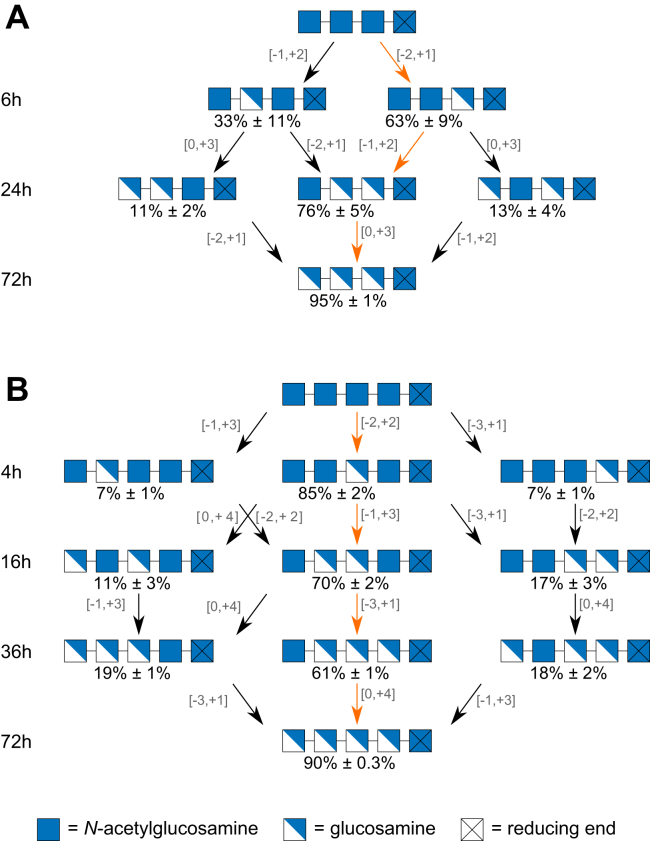
**AngCDA deacetylating COS.***A*, mode of action of AngCDA deacetylating chitotetraose (A4). *B*, mode of action of AngCDA deacetylating chitopentaose (A5). Products detected in relative amounts below 5% and 7% are not shown for A4 and A5, respectively. Times shown on the *left* indicate at which time points the samples were taken (Fig. 1). *Orange arrows* indicate the main path. The binding mode for each step is indicated next to the corresponding *arrow* (n = 3). COS, chitooligosaccharide.

To validate the conclusions drawn from the MD simulations and to gain a better understanding of how AngCDA deacetylates and *N*-acetylates its substrates, the enzyme was incubated with A4, A5 (as shown in Fig. 1), and D4. For A4 and A5, samples were taken at those time points where the different products had the highest relative concentration, to determine their PA and, thus, the mode of action of the enzyme (Fig. 7). In both acetylated substrates, the internal units were deacetylated first, step by step generating ADDA and ADDDA, before finally DDDA and DDDDA were produced, as previously described for other CDAs such as AnCDA and ArCE4A (33, 34). While AngCDA prefers to deacetylate the third unit from the nonreducing end in both substrates first, this preference seems to be more pronounced for A5, resulting in AADAA in rather pure form. To our knowledge, this makes AngCDA the first CDA to mainly produce this acetylation pattern. On closer inspection of the A4 deacetylation, the comparably high standard deviation for A3D1 suggests that the exact time point influences which products are present. If the reaction was slightly less advanced, more AADA was found, whereas later, ADAA seemed to accumulate as further discussed in the Activity on defined A3D1 paCOS section.

**Figure 8:**
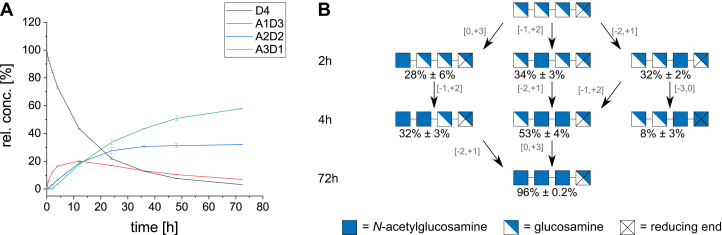
**AngCDA** ***N*****-acetylating COS.***A*, product development over time of AngCDA *N*-acetylating GlcN-tetraose (D4). *B*, mode of action of AngCDA *N*-acetylating D4. Products detected in relative amounts below 7% are not shown. Times shown on the *left* indicate at which time points the samples were taken during the reaction shown in *A*. The binding mode for each step is indicated next to the corresponding *arrow* (n = 3). COS, chitooligosaccharide.

In contrast to the deacetylation of fully acetylated COS (forward mode, Figs. 1 and 7), the *N*-acetylation of GlcN-tetraose (D4) (reverse mode, Fig. 8) looks rather different. While deacetylating, AngCDA generates products with decreasing DA in succession (see Sequence analysis and initial protein characterization section), whereas during the *N*-acetylation of D4, none of the intermediate products show a distinct peak (Fig. 8*A*). As no clear peaks were visible for the intermediates regarding their DA, the PA of these products was determined at early time points during the reaction (Fig. 8*B*). No clear preference can be observed for the first *N*-acetylation, where any sugar unit except for the reducing end unit was *N*-acetylated to an equal extent. Then, the neighboring unit toward the reducing end was *N*-acetylated, apparently also *N*-acetylating the reducing end unit itself. However, as the final product A3D1 clearly consisted of mainly AAAD, the DDAA detected might represent an artifact from the difficult sequencing of the low amounts of A2D2 at early time points.

All in all, for both deacetylation and *N*-acetylation, AngCDA seems to prefer substrates with a higher DA. In forward mode, first the substrate itself and then the following products are preferably deacetylated, whereas the enzyme avoids placing a deacetylated unit at subsite −1. In reverse mode, the first products are preferably *N*-acetylated further, presumably placing the already acetylated unit at subsite −1. To further investigate these subsite preferences, the AngCDA activity on the four different defined A3D1 paCOS were tested.

### Activity on defined A3D1 paCOS

**Figure 9:**
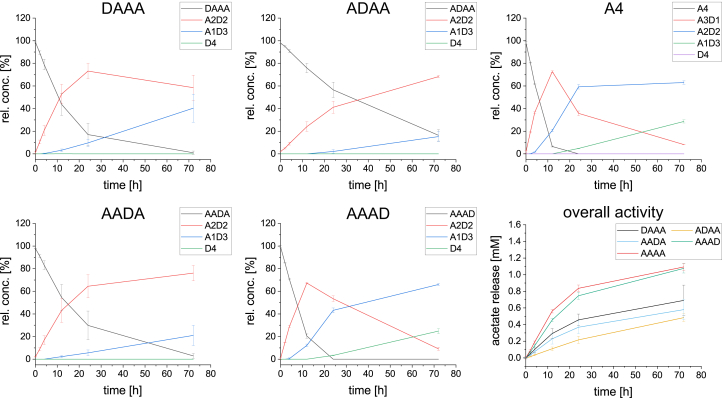
**Product development over time using different A3D1 paCOS.** Graphs DAAA, ADAA, AADA, AAAD, and A4 show the relative substrate (0.5 mM) and all product concentrations for a 72-h reaction period. The final graph gives the overall activity in terms of the calculated total acetate release. Since this is an independent experiment with a different enzyme concentration, the activity on A4 is slightly different from that shown in Figure 1 (n = 3). paCOS, partially acetylated chitooligosaccharide.

AngCDA was incubated with the four monodeacetylated paCOS DAAA, ADAA, AADA, and AAAD in direct comparison to A4, and the substrate and product concentrations were monitored during the deacetylation reaction (Fig. 9). Only the product development on AAAD was similar to that on A4. The other substrates were deacetylated at lower rates, with ADAA showing the slowest acetate release with only 0.5 mM after 72 h, corresponding to one deacetylation per substrate, whereas these levels were reached for A4 already within the first 12 h. As expected from the MD simulations and the activity assay on D4, partially deacetylated substrates are not preferred by AngCDA. However, the position of the GlcN unit seems to play a major role. If the reducing end of the substrate is a deacetylated GlcN unit, a position that cannot easily be deacetylated by AngCDA, the enzyme is still able to deacetylate the remaining three units, that is, the ones it also deacetylates in A4 (Fig. 7*A*). For all the other monodeacetylated substrates (DAAA, ADAA, and AADA), the GlcN unit is in a position at which it would be deacetylated by AngCDA in A4. Since in the observed period, only up to two units were deacetylated, it could have been assumed that the activity on DAAA should be comparable to the activity on A4, as the first two deacetylations on A4 occur at the internal units only. However, this was not the case. The reduced activity on DAAA compared with A4 suggests a preference for a GlcNAc unit at either or both subsite −1 and/or −2. This would also explain the strongly reduced activity on ADAA, since according to the mode of action (Fig. 7*A*), it is quite likely that the GlcN unit in this substrate would be placed at subsite −1. This would also explain why ADAA accumulated when AngCDA deacetylates A4, as the other monodeacetylated product, AADA, is a preferred substrate for the second deacetylation step. Indeed, this was also observed here, when AngCDA was more active on AADA compared with ADAA.

## Discussion

We have performed a more in-depth *in vitro* and *in silico* analysis on AngCDA than previously reported for any other CDA. Concerning the pH and temperature optima, activity on chitin and chitosan polymers and activity on COS DP1-6, AngCDA is similar to already described CDAs (26, 27, 33, 34). We thus assume that our insight into this fungal CDA allows a deeper understanding of other CDAs of both bacterial and fungal origin.

For the bacterial VcCDA and the fungal PcCDA, critical loops were identified that shape the substrate-binding site and, thus, determine the substrate-binding mode and, consequently, the PA of the products generated (22, 28). According to the subsite capping model, these loops block parts of the binding site, forcing the substrate to bind in a particular position and preventing deacetylation of polymeric substrates. CDAs such as the fungal AngCDA, AnCDA, and ClCDA as well as the bacterial ArCE4A, which are active on polymeric substrates, have much smaller loops that do not block parts of the binding site, leaving it more open and accessible for polymers (32, 34, 33). Nonetheless, their corresponding smaller loops form the majority of the binding site and most likely contribute to the different modes of action observed for these CDAs. Based on recent results (12), we assume that in addition, and probably even more dominantly in the case of the more open CDAs, the preference for acetylated or deacetylated sugar units at the different subsites of the enzymes also contribute to defining the PA of the products. To our knowledge, clear subsite preferences have so far only been described for ClCDA (reporting a preference for GlcNAc at subsite −2) (21) and CnCDA4 (reporting a preference for GlcN at subsite −1) (2). For other CDAs, such as PesCDA and PgtCDA, such preferences can only be presumed based on the available data (12).

### Comparison between best binding modes *in silico* and *in vitro*

To obtain deeper insights into substrate binding and possible preferences for acetylated or deacetylated units at the different subsites of AngCDA, we performed *in vitro* studies of the mode of action on fully acetylated substrates A4 and A5, on the four monodeacetylated substrates A3D1, and on the fully deacetylated substrate D4, the latter in reverse *N*-acetylation mode, and compared the results with the detailed *in silico* analysis focusing on the same substrates. Based on the *in silico* data, we would have expected six subsites ranging from −3 to +2, but we did not observe a significantly faster first deacetylation of A6 than of A5. And contrary to the binding energy calculated for the three different binding modes of A5, where the lowest energy was calculated for binding mode [−3, +1], the mode of action shows that the middle unit is deacetylated first, meaning that the preferred binding mode is in fact [−2, +2]. This hints at a minor role for subsite −3, if any. Similarly, the RMSF values, which are lowest for binding mode [−3, 0] and [−3, +1] for A4 and A5, respectively, suggests a stable binding in these orientations, but the corresponding products AAAD and AAADA were not or only rarely observed *in vitro*.

It is important to highlight that (i) before the start of the simulations, the substrates were already positioned in their binding modes and (ii) standard MD simulations are not able to simulate the catalytic reaction. Thus, either the entry of the substrate into the binding site or the reaction itself might account for the differences between best binding modes observed *in vitro* and *in silico*. Since the *in silico* data suggest that the sugar unit at subsite 0 is still correctly positioned for deacetylation in binding modes ending at subsite 0, we expect that the substrate entry into the binding site might have a higher energy barrier for binding modes mainly including the minus subsites. As already reported or suggested for other CDAs, the +1 subsite might play an important role for efficient catalysis, as it appears to be necessary for substrate binding. For ArCE4A, the only CDA with an open binding site that has been crystallized with its substrate, only two units of the chitotetraose substrate were resolved, at subsites 0 and +1, indicating a very stable binding at these positions only (33). And for all CDAs classified in the CE4 family, at least two sugar units are needed for activity (2, 4, 26, 27, 33, 34). The importance of subsite +1 is also visible for AngCDA in the binding free energy calculations, where, besides the sugar unit in subsite 0, the sugar unit at subsite +1 shows the lowest binding energy. The most consistently observed hydrogen bond at subsite +1 involves Asp162, which is also highly conserved in other CDAs (first D in motif 4, see Fig. 2). It should be noted here that this hydrogen bond was often present at the beginning, while it tended to break at some point during the simulation and was rarely reestablished. This may indicate that it is primarily important for substrate entry and may play a minor role in keeping the substrate in the binding site, explaining the differences observed between *in silico* and *in vitro* results. Furthermore, the energy contribution of the zinc ion is highly positive in all simulations, suggesting that the zinc ion prefers to be surrounded by water instead of interacting with the substrate. In conclusion, we thus hypothesize that the substrate first interacts with Asp162 at subsite +1 and possibly with Lys164 at subsite +2, allowing it to then displace the water molecules surrounding the zinc ion.

### Subsite preferences for GlcNAc and GlcN units

In addition to hydrogen bonds, which contribute to substrate binding and possibly substrate entry, other interactions certainly play a role as well. Hydrophobic interactions between Phe139 and Leu193 on the one hand and the acetyl methyl group on the other hand strongly contribute to the substrate binding at subsite 0. Stacking interactions between aromatic residues, such as Tyr138 and Tyr166, further contribute to a strong binding at subsite 0. Only salt bridges were not observed *in silico*, as all substrates were uncharged. Given that the MD simulations do not cover the substrate entry into the binding cleft, as discussed previously, the preferred binding mode is difficult to predict from these simulations. Nonetheless, they provide valuable insights into the subsite preferences, namely a strong preference for a GlcNAc unit at subsite −1 and probably weak preferences for GlcNAc units at the other subsites. But even with these detailed simulations, it remains difficult to develop a hypothesis that would clearly explain these GlcNAc preferences. The main difference between A4 and ADAA in binding mode [−2, +1] is the rotation of the GlcN unit at subsite −1 resulting in a completely different orientation of the neighboring nonreducing end unit. This is enabled by the missing acetyl group and residues Thr197 and Tyr138, which seem to stabilize this orientation (Fig. 4). However, compared with the A4 simulations where the positioning of the GlcNAc unit at subsite −1 is highly reproducible, this reorientation of the GlcN unit is not visible in all ADaA trajectories. This would suggest that contrary to a GlcN unit, a distinct energy minimum for a GlcNAc unit exists at subsite −1, resulting in a GlcNAc preference. A weak GlcNAc preference at subsite +1, deduced from both the *in silico* and *in vitro* results, could be attributed to Asn167, which occasionally formed a hydrogen bond with the acetyl oxygen but did not interact with a GlcN unit (see the hbonds occupancy spreadsheet in the supporting information). At subsite −2, Leu73 might contribute to a weak preference for acetylated units, but this preference is difficult to confirm with our *in vitro* results. As proposed by Wattjes *et al*. (12), these subsite preferences are thought to influence the acetylation pattern generated especially on polymeric substrates. They describe *N*-acetylation of fully deacetylated polyglucosamine polymers using different CDAs including the here characterized *A. niger* CDA. Their results show that AngCDA (named AnCDA in their article) generates a more blockwise acetylation pattern, preferably *N*-acetylating neighboring units of already acetylated ones. Our results suggest that this *N*-acetylation occurs toward the reducing end.

## Conclusion and outlook

Our results show that the combination of *in silico* and *in vitro* methods can unveil more details regarding the mechanisms underlying the generation of different acetylation patterns by CDAs. On the one hand, without simulating substrate entry into the active site, the *in silico* predictions concerning the most favored binding mode need to be validated experimentally and evaluated carefully. On the other hand, the computational comparison between COS and paCOS seems to open the door for a detailed analysis of subsite preferences in CDAs, which would be very tedious in the laboratory. However, given the current state of the art, any conclusions from the *in silico* data will require final *in vitro* validation by testing the activity on different defined paCOS selected based on the computational results. As a further proof of concept, the *in silico* approach introduced here should be tested with other CDAs, such as the chitosan deacetylase CnCDA4 with its uniquely strong preference for GlcN at its −1 subsite (2).

While the acetylation pattern generated on smaller oligomers seems to be defined by the available and accessible subsites, it appears to be strongly influenced by subsite preferences when acting on larger oligomers or on polymeric substrates. While CDAs were reported to retain their regioselectivity on A4 and D4 in forward and reverse mode, respectively (23), this does not appear to be the case for polymers. Here, a GlcN preference next to subsite 0, that is, at either subsite −1 or +1, leads to an alternating or blockwise acetylation pattern when the substrate is *N*-acetylated or deacetylated, respectively (see studies on PgtCDA (12, 17)).

Based on these recent findings and our results presented here, we suggest that any detailed characterization of a CDA needs to include a thorough analysis of its subsite preferences, like in the case of chitinases and chitosanases, which can even be classified based on their subsite specificities and preferences (37). A CDA like AngCDA, which apparently favors GlcNAc at all subsites, is expected to produce a random acetylation pattern when deacetylating high DA chitosans and rather large GlcNAc blocks when *N*-acetylating polyglucosamine. A hypothetical CDA, with a different preference at subsite +1 (*e.g.*, a GlcNAc preference at subsite −1 and a GlcN preference at subsite +1) would presumably produce small GlcN or GlcNAc blocks when deacetylating or *N*-acetylating the substrate, since single GlcNAc or GlcN units between these blocks would not be further deacetylated or *N*-acetylated, respectively.

Our combined *in vitro* and *in silico* approach can help to elucidate these subsite preferences. The detailed understanding thus gained of the most influential residues can then be used for protein engineering to tailor the subsite preferences, improving access to a broader range of chitosan oligomers with fully defined architecture and of chitosan polymers with defined nonrandom patterns of acetylation. Such third-generation chitosans are a prerequisite to understand the physiological roles of CDA-generated PAs in natural chitosans, and they are promising the next breakthrough in the development of reliable chitosan-based applications, for example, in agriculture or biomedicine.

## Experimental procedures

### In silico

#### Sequence analysis

The AngCDA sequence (UniProt ID: A2QZC8) was analyzed using several online tools including the conserved domain databank (38, 39) for domain prediction, SignalP-5.0 (40) for signal peptide prediction, PredGPI (41) and NetGPI-1.1 (42) for GPI anchor prediction, and TMHMM (43) for transmembrane region prediction.

#### Multiple structure alignment

To our knowledge, the crystal structures of eight CE4 enzymes acting on chitinous substrates are described so far, including AnCDA (Protein Data Bank [PDB]: 2Y8U) (34), ArCE4A (PDB: 5LFZ) (33), ClCDA (PDB: 2IW0) (32), SlCE4 (PDB: 2CC0) (35), VcCDA (PDB: 4NY2) (22), SpPgdA (PDB: 2C1G) (44), BsPdaC (PDB: 6H8L) (27), and BmCDA8 (PDB: 5Z34) (29). All of them, except for BmCDA8, were used for a multiple structure alignment using the PyMOL plugin PyMod3 with SALIGN (45, 46). If multiple chains were present in the crystal structure, only chain A was used for the alignment. For ClCDA, the His-tag was removed; for VcCDA, the chitin-binding domains (residues 336–433); and for SpPgdA, the two additional domains (residues 46–266) were deleted from the structure. The 3D structures were colored by conservation with the CAMPO score (47) using the Blosum62 scoring matrix (48).

#### Ligand generation and docking

The 3D structures of chitotetraose (A4) and chitopentaose (A5) were created using the carbohydrate builder from GLYCAM Web (www.glycam.org/cb) (49). To create ligands with deacetylated units, the acetyl group was removed using the builder function in PyMOL (45), and the residues and atom names were adjusted to fit the GLYCAM names (50).

For docking, the ligands (A4, A5, DAAA, ADAA, AADA, and AAAD) and the receptor (AngCDA crystal structure) were prepared using the prepare_ligand4.py and prepare_receptor4.py scripts included in the AutoDockTools (51). The histidine protonation was set to HID for the metal coordinating His97 and His101. The charge of the zinc ion was set to +2. Docking was performed using AutoDock VinaCarb v1.0 with the default parameters for chi_coeff and chi_cutoff suggested by the authors (52, 53).

#### MD simulations

All simulations were run with the GROMACS 2019 package (54, 55, 56, 57, 58, 59, 60, 61) using the Amber force field ff14SB (62) for the protein, the compatible GLYCAM06 force field (50, 63) for the ligands, and TIP3P as the water model (64). The histidine protonation was set to HID for the metal coordinating His97 and His101 and to HIP for the catalytic His195. Since the GLYCAM06 force field is not available in the GROMACS package, the ligands were first prepared using the LEaP program included in AmberTools20 (65) and then converted into GROMACS format using the GLYCAM06-compatible ACPYPE (66, 67). Water molecules were added in a dodecahedron-shaped box with a distance of 10 Å to each side. The charge was neutralized with sodium ions before running the energy minimization using steepest descent. Before the production run, NVT and NPT equilibrations were conducted using the leap-frog integrator in 2 fs steps for 100 ps with a V-rescale thermostat at 310 K and a Berendsen barostat at 1 bar. The same integrator and step size were used for the final NPT production run, using a V-rescale thermostat at 310 K as well, but the more precise Parrinello–Rahman barostat at 1 bar. A Verlet cutoff scheme was used for van der Waals interactions, and the Ewald summation was used for long-range electrostatic interactions. Distance restraints between the metal ion and the metal-binding triad (D48, H97, and H101), the catalytic aspartate (D47), and the catalytic water molecule and a dihedral restraint of the catalytic aspartate (D47) were applied to stabilize the metal binding during the simulation.

The resulting trajectories were analyzed using the GROMACS-implemented RMSF tool to detect the fluctuation of the ligand in the active site after the complex was centered in the simulation box and rotational and translational movements of the complex were removed. These trajectories were inspected with vmd (68), and the vmd hbond plugin 1.2 was used with a distance cutoff of 3.5 nm and an angle cutoff of 35° to detect all hydrogen bonds between the enzyme and the ligand during the simulations. In addition, the molecular mechanics generalized Born surface area approach was applied to calculate the average binding energy of the ligand throughout the whole trajectory using gmx_MMPBSA (69), a GROMACS implementation of MMPBSA.py (70) from AmberTools20 (65). The GB implicit solvent model was used in a single trajectory approximation using standard parameters (igb = 5, saltcon = 0.150) as described in the online documentation. All docked A4 substrates were chosen to find any residue within 6 Å around the substrates, and these residues were used for a decomposition analysis of all trajectories.

Representative structures for a visual comparison (Fig. 4) were generated using the Jarvis–Patrick clustering algorithm implemented in GROMACS. First, one snapshot was taken from the main cluster of each trajectory, which is most similar to all other snapshots from this cluster. Then, these snapshots from the nine replicates were aligned, and again, the one most similar to all others was used as a representative structure for the corresponding substrate and binding mode.

### In vitro

#### Cloning

The AngCDA gene (UniProt ID: A2QZC8), without the sequence encoding the 19 amino acid signal peptide, was codon optimized for *Pichia pastoris* and synthesized by GeneArt. It was amplified with corresponding overlaps to be cloned *via* Gibson assembly (71) into a previously generated pET-22b(+) plasmid (Merck KGaA), already containing either an N-terminal pelB and a C-terminal Strep-tag II sequence or both an N- and C- terminal Strep- tag II. Both constructs were transformed into *E. coli* Rosetta™ 2 (DE3) cells (Merck KGaA) for protein expression.

#### Protein expression and purification

For both constructs, *E. coli* Rosetta™ 2 (DE3) cells carrying the desired plasmid were grown in 500 ml LB auto-induction medium as described by Studier (72) at 26 °C (for activity tests) or 30 °C (for protein crystallization) for 48 h. Then, cells were harvested at 4000*g* for 20 min at 4 °C, resuspended in 20 to 30 ml FPLC washing buffer (20 mM TEA, 400 mM NaCl, and pH 8) and stored at –20 °C. For protein crystallography, lysozyme (1.5 g/l final concentration) and NaCl (10 g/l final concentration) were added, and the cells were thawed at RT before they were further lysed by five 15-s pulses at 40% amplitude using a Branson Digital Sonifier model 250-D (Emerson). For all activity assays, the cells were thawed at RT before 3 μl benzonase (Merck KGaA, 25 U/μl) in 250 μl 2 M MgCl_2_ were added and incubated at RT for 15 min, shaking slightly. Then, 2 ml high salt buffer (1 M TEA, 1 M NaCl, and pH 8) were added before the cells were lysed by sonication as described previously. For both objectives, the lysed cells were centrifuged for 60 min at 40,000*g* at 4 °C, and the AngCDA was purified from the supernatant by affinity chromatography using the Strep-TactinXT purification system (IBA). Finally, the enzymes were concentrated with Amicon Ultra-15 centrifugal filters (Merck KGaA) and rebuffered into Tris–HCl, pH 8, and 100 mM NaCl for protein crystallization or into 50 mM TEA, pH 7, for activity assays.

#### Protein crystallization

For further purification before crystallization, a size-exclusion chromatography was performed at 20 °C on a Superdex 75 16/600 column (GE Healthcare) equilibrated with 50 mM Tris–HCl, pH 8, and 100 mM NaCl. Peak fractions corresponding to the AngCDA monomer were pooled and concentrated with an Amicon Ultra-15 centrifugal filter to 4.57 mg/ml before the crystallization screening. Crystals of AngCDA were found in sitting-drop vapor-diffusion experiments at 20 °C. Drops were prepared by mixing 0.2 μl of AngCDA and 0.1 μl of reservoir containing 1.4 M sodium malonate and 0.1 M Bis–Tris propane at pH 7. Crystals were cryoprotected by soaking in 10% (v/v) glycerol in crystallization buffer, before flash freezing in liquid nitrogen. Diffraction data were collected at 100 K at microfocus beamline Proxima-2A (Soleil). The collected images were processed using XDS (73) and scaled in the CCP4 suite program AIMLESS (74). MOLREP (75) was used for molecular replacement using *A. nidulans* CDA (AnCDA, PDB: 2Y8U) (34). The structure was rebuilt with Coot (76) and refined with PHENIX (77). The final model was evaluated with MolProbity (78). All values obtained and generated are shown in Table 1.

**Table 1:** Data collection and refinement statistics for the crystal structure of the chitin deacetylase of *Aspergillus niger* CBS 513.88

Data collection	AngCDA
Beam line	PROXIMA-2A
Space group	*P*432
Average unit cell (Å)	a = b = c = 119.87
Wavelength (Å)	0.98012
Resolution (Å)	48.94–1.81 (1.85–1.81)
R_pim_	0.043 (0.771)
No. of unique reflections	27,485 (1613)
Mean I/σI	18.6 (1.9)
CC_1/2_	0.999 (0.716)
Completeness (%)	100.0 (100.0)
Average redundancy	77.5 (79.6)
Refinement	
Resolution (Å)	42.38–1.81
R_free_/R_work_	18.55/15.26
Total number of atoms	1965
Water	237
Average *B*-factor	26.56
Ligands	ZN; MLI; CL
RMSD	
Bonds	0.006
Angles	0.889
MolProbity analysis	
Clashscore, all atoms	0.29 (100%)
MolProbity score	0.86 (100%)
PDB entry	7BLY

Values in parentheses refer to the outer resolution shell.

#### pH and temperature optimum

The enzyme activity was tested from pH 2 to 12 in increments of 1 and at pH 7.5 and 8.5 using 50% (v/v) Teorell Stenhagen buffer (79, 80) with 18.975 μg/ml purified AngCDA and 0.5 mM A4. In addition, the activity was also tested for 50 mM TEA buffer at pH 7. After 30 min of incubation at 37 °C, the reaction was stopped with one volume 0.1 M HCl, and the products were measured *via* hydrophilic liquid interaction chromatography (HILIC)–electrospray ionization–MS as described in the Activity assays on oligomeric substrates section.

#### Activity assay on polymeric substrates

Chitosan with DA 0% as well as α-chitin or β-chitin was kindly provided by Dominique Gillet, Gillet Chitosan. The DA 0% chitosan was *N*-acetylated to DA 12%, 32%, and 46% according to Lamarque *et al.* (15). The DA was analyzed using 400 MHz 1H NMR (81), and the DP and dispersity (DP 1600 and Đ 1.88 for the starting material) were analyzed using SEC-RI-MALLS (17, 82). Colloidal chitin was prepared as described by Hsu *et al.* (83).

To determine the activity on these substrates, 18.975 μg/ml purified AngCDA was incubated with 1 mg/ml substrate in 50 mM TEA buffer (pH 7) at 30 °C for 24 h. Samples were taken after 2, 4, and 24 h, and the reaction was stopped with one volume of 66.6 mM HCl. Samples containing insoluble chitin substrates were shaken during incubation to avoid precipitation of the substrates. The released acetate was quantified with the acetic acid kit from R-Biopharm using reduced volumes (100 μl sample, 63 μl water, 100 μl solution 1, 20 μl solution 2, 20 μl 1:20 diluted solution 3, and 20 μl 1:10 diluted suspension 4) to fit in a microtiter plate. The Δacetic acid was calculated as described in the kit. A standard curve was generated with 0.05, 0.03, 0.015, and 0.003 g/l acetic acid to calculate the acetate concentration in each sample, which directly correlates with the ΔDA.

#### Activity assays on oligomeric substrates

Oligomeric substrates chitobiose (A2), chitotriose (A3), and chitohexaose (A6) were purchased from Megazyme. Chitosantetraose (D4) was purchased from Biosynth. A mixture of freeze-dried chitotetraose (A4) and chitopentaose (A5) was kindly provided by the Bio Base Europe Pilot Plant, produced as described by Hamer *et al.* (24). The mixture was dissolved in 1:1 H_2_O:ACN (15 mg/ml) and purified *via* HILIC using the modular LC-20A Prominence HPLC system (Shimadzu Deutschland GmbH) with a BEH Amide column (5 μm, 10 × 250 mm; Waters Corporation). A gradient shifting from solvent A (80% [v/v] ACN, 20% [v/v] H_2_O) to solvent B (80% [v/v] H_2_O, 20% [v/v] ACN), both with 10 mM NH_4_HCO_2_ and 0.1% (v/v) formic acid, was used to separate the two oligomers. Collected fractions were freeze dried and dissolved in a final concentration of 10 mM. A3D1 substrates were generated by incubating A4 with NodB (DAAA) (24), VcCDA (ADAA), (22) or PesCDA (AADA) (4) in 50 mM TEA buffer (pH 7) or by incubating D4 with AngCDA (AAAD) in 2.25 M NaAc buffer (pH 7) at 37 °C. All samples were freeze dried, and the paCOS generated were separated as described for A4 and A5.

For all tests, 0.5 mM substrate was incubated at 37 °C, pH 7 either using 50 mM TEA buffer for deacetylation or 2 M NH_4_Ac for *N*-acetylation. The deacetylation and *N*-acetylation reactions were stopped with one volume of 1% formic acid or by freezing the samples, respectively. The enzyme concentration was adjusted to an activity resulting in 10 to 20% A3D1 after 30-min incubation with A4 for deacetylation reactions and in 50 to 60% A3D1 after 72-h incubation with D4 for *N*-acetylation reactions.

All paCOS generated from these activity assays were chemically *N*-acetylated with acetic anhydride-d_6_ (Merck KGaA) to allow quantification by MS analysis as described by Cord-Landwehr *et al*. (84). To this end, the samples were freeze dried and resolved in 10 μl H_2_O before 10 μl 100 mM NaHCO_3_ were added, followed by 20 μl methanol with 1 μl acetic anhydride-d_6_. After 30 min of incubation at 30 °C and 1200 rpm, 10 μl methanol with 1 μl acetic anhydride-d_6_ were added before a second 30-min incubation under the same conditions. Finally, the samples were freeze dried again and resolved in their initial volume for quantitative MS analysis according to Hamer *et al*. (24).

#### Sequencing of A4, A5, and D4 products

To determine the PA of paCOS generated by A4 and A5 deacetylation, samples corresponding to the time points with the highest concentration of the respective paCOS were chosen (*e.g.*, 6 h for A3D1, see Fig. 1). As products generated from D4 *N*-acetylation did not peak one after the other, early time points were chosen for A1D3 and A2D2 pattern analyses to avoid the influence of isotope peaks from the following products. The pattern was determined as described by Cord-Landwehr *et al*. (84). To this end, the samples were chemically *N*-acetylated as described previously, before reducing end labeling was performed in two steps with 3 and 10 μl H_2_^18^O at 70 °C for 3 and 18 h, respectively. Finally, the respective paCOS for the chosen time points were analyzed *via* HILIC–electrospray ionization–MS (2), and fragment intensities were used to determine the PA.

## Data availability

Data that are not included in the main article can be found in the supporting information pdf document and the two spreadsheets. The crystal structure of AngCDA can be found in the Protein Data Bank with the ID 7BLY.

## Supporting information

This article contains supporting information.

## Conflict of interest

The authors declare that they have no conflicts of interest with the contents of this article.
