# Supplementary material for: In silico and in vitro analysis of an Aspergillus niger chitin deacetylase to decipher its subsite sugar preferences
Source: J Biol Chem. 2021 Sep 1;297(4):101129. doi: 10.1016/j.jbc.2021.101129 (PMC8488497; doi:10.1016/j.jbc.2021.101129)
Supplement: Figures S1–S6 and Tables S1–S5 [file mmc1.pdf]

Supporting information

In silico and *in vitro* analysis of an *Aspergillus niger* chitin deacetylase to decipher its subsite sugar preferences

M. Bonin, L. Hameleers, L. Hembach, T. Roret, S. Cord-Landwehr, G. Michel, B. M. Moerschbacher

List of Figures

S1 Results from the conserved domain databank S-1
S2 Results from SignalP-5.0 . . . . . S-2
S3 Results from NetGPI and PredGPI S-2
S4 Results from TMHMM S-3
S5 Temperature and pH optimum of AngCDA S-5
S6 AngCDA activity on polymeric substrates S-5

List of Tables

S1 Docking scores of all substrates and binding modes S-3
S2 Identity values for different CE4 enzymes S-4
S3 Root mean square deviation for different CE4 enzymes S-4
S4 Energy contribution of all active site residues S-4
S5 Hydrogen bond occupancy of all substrates in all binding modes S-4

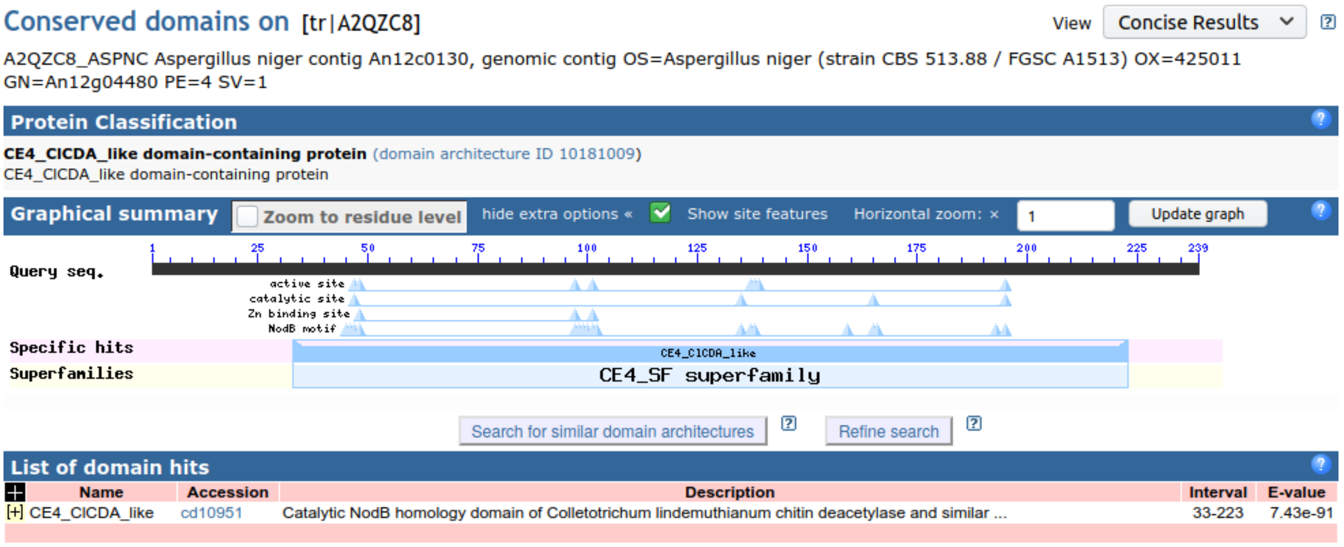

Figure S1: Result from the conserved domain databank (https://www.ncbi.nlm.nih.gov/cdd/) for the AngCDA sequence (UniProt ID: A2QZC8). A CE4_ClCDA_like and CE4_SF superfamily domain was detected from residues 33 to 223.

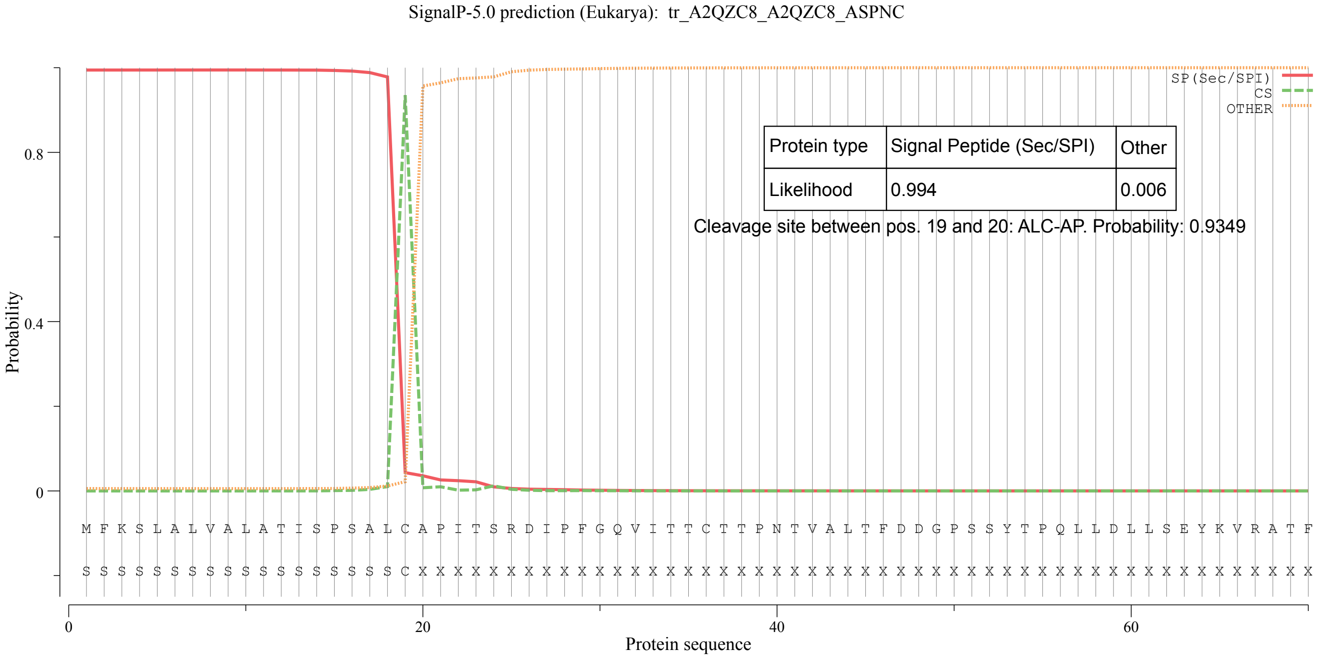

Figure S2: Result from SignalP-5.0 (http://www.cbs.dtu.dk/services/SignalP/) signal peptide predictionfor the AngCDA sequence (UniProt ID: A2QZC8). A signal peptide was predicted until residue 19 with a likelihood of 0.994.

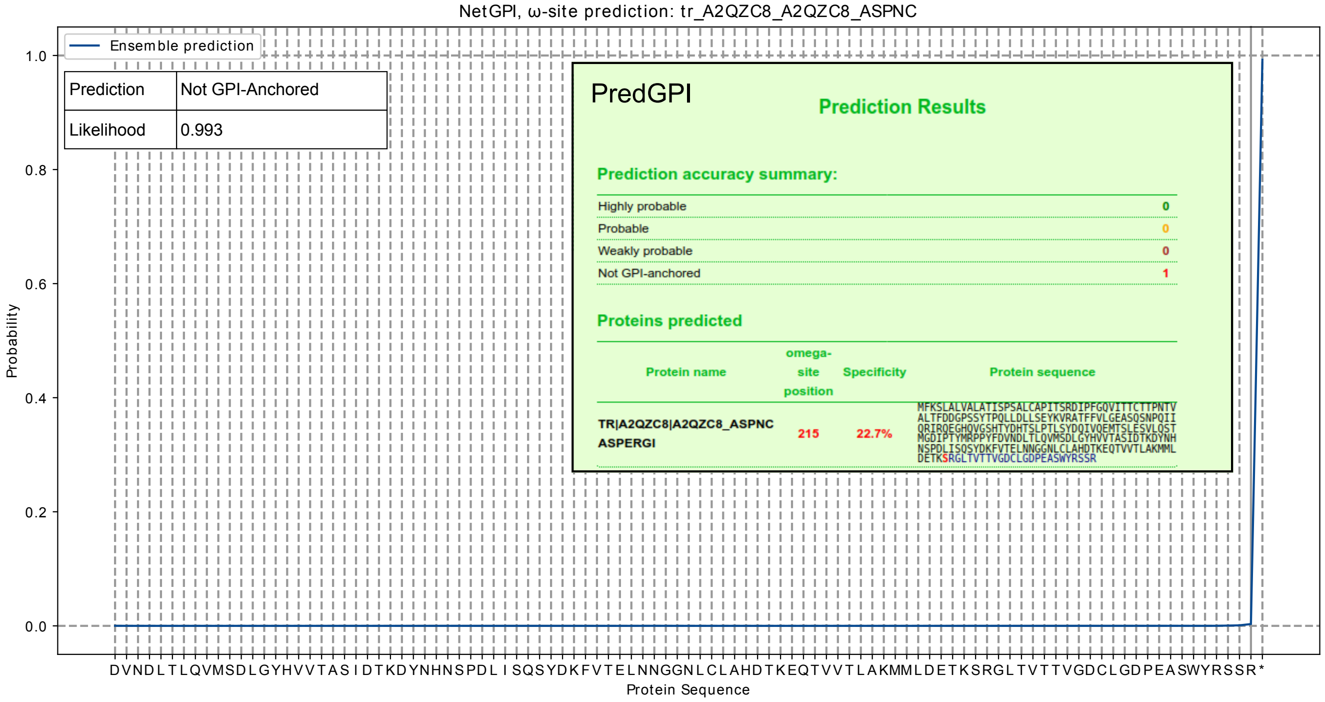

Figure S3: Result from NetGPI1.1 (https://services.healthtech.dtu.dk/service.php?NetGPI-1.1) (outer graph) and PredGPI (http://gpcr.biocomp.unibo.it/predgpi/) (inner green box) GPI anchor prediction for the AngCDA sequence (UniProt ID: A2QZC8). Both predicted, that the AngCDA is not GPI-anchored.

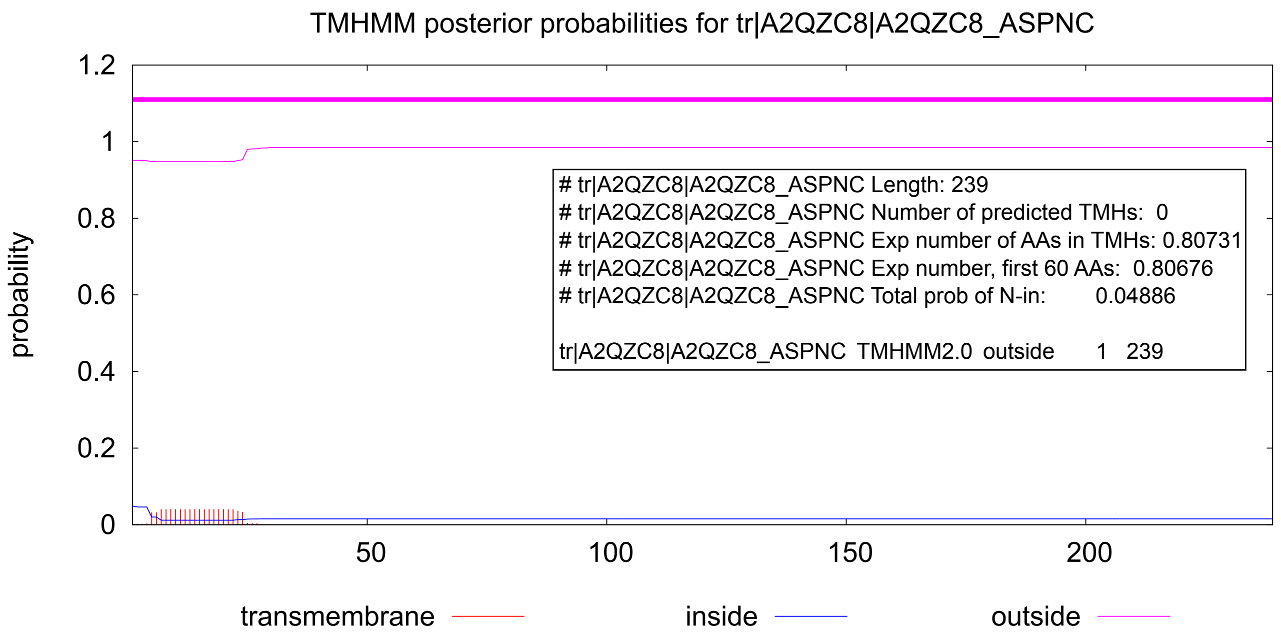

Figure S4: Result from TMHMM (http://www.cbs.dtu.dk/services/TMHMM/) transmembrane prediction for the AngCDA sequence (UniProt ID: A2QZC8). The complete protein was predicted as an outside protein without any transmembrane region.

Table S1: Docking scores of all binding modes generated for molecular dynamics simulations. The first column indicates the substrate, while the second column indicates the binding mode with a small letter representing the sugar bound at subsite 0.

Substrate		docking scores (kcal/mol)		
		conf 1	conf 2	conf 3
A4	aAAA	-6.3	-6.1	-5.9
	AaAA	-7.2	-7.1	-6.1
	AAaA	-6.6	-6.5	-6.0
	AAAa	-6.2	-5.8	-5.4
A5	AaAAA	-6.9	-6.4	-6.3
	AAaAA	-7.1	-6.8	-6.3
	AAAaA	-6.8	-6.7	-6.5
AAAD	aAAD	-6.4	-6.4	-6.0
	AaAD	-7.0	-6.9	-6.5
	AAaD	-6.9	-6.8	-6.1
AADA	AaDA	-7.2	-7.1	-6.9
	AAdA	-5.9	-5.8	-5.1
ADAA	AdAA	-6.9	-5.9	-5.8
	ADaA	-7.0	-6.7	-6.4
DAAA	DaAA	-6.5	-6.4	-5.6
	DAaA	-6.1	-6.1	-6.1
	DAAa	-5.4	-5.3	-4.9

Table S2: Identity values for the different CE4 enzymes used in the alignment shown in figure 2.

Identity [%]	AngCDA	AnCDA	ClCDA	ArCE4A	SpPgdA	BsPdaC	SlCE4	VcCDA
AngCDA	100	60.95	44.6	34.59	32.97	30.43	26.63	19.79
AnCDA	60.95	100	45.24	37.1	35.52	31.89	32.97	23.4
ClCDA	44.6	45.24	100	32.97	35.16	28.26	28.8	22.8
ArCE4A	34.59	37.1	32.97	100	35.57	40.21	30.43	21.79
SpPgdA	32.97	35.52	35.16	35.57	100	46.49	32.97	28.65
BsPdaC	30.43	31.89	28.26	40.21	46.49	100	32.43	23.46
SlCE4	26.63	32.97	28.8	30.43	32.97	32.43	100	20.99
VcCDA	19.79	23.4	22.8	21.79	28.65	23.46	20.99	100

Table S3: Root mean square deviation (rmsd) for the different CE4 enzymes used for the alignment shown in figure 2.

rmsd [Å]	AngCDA	AnCDA	ClCDA	ArCE4A	SpPgdA	BsPdaC	SlCE4	VcCDA
AngCDA	-	0.498	1.1703	1.6278	1.3974	1.9251	2.209	2.8473
AnCDA	0.498	-	1.2041	1.7002	1.4866	1.9564	2.2549	2.9485
ClCDA	1.1703	1.2041	-	1.5103	1.3878	2.0001	2.2227	2.998
ArCE4A	1.6278	1.7002	1.5103	-	1.1688	1.5369	2.2278	2.7168
SpPgdA	1.3974	1.4866	1.3878	1.1688	-	1.4632	1.9319	2.5988
BsPdaC	1.9251	1.9564	2.0001	1.5369	1.4632	-	2.2126	2.7197
SlCE4	2.209	2.2549	2.2227	2.2278	1.9319	2.2126	-	2.8549
VcCDA	2.8473	2.9485	2.998	2.7168	2.5988	2.7197	2.8549	-

Table S4: A small section of the energy contribution of all active site residues and all sugar units for all simulations. The complete table is included in the supporting material as an additional spreadsheet.

Substrate →		A4							
binding mode →		aAAA		AaAA		AAaA		AAAa	
residue	number (subsite)	total average	total StDev	total average	total StDev	total average	total StDev	total average	total StDev
VAL	32 (+3)	0.02	0.00	0.02	0.00	0.02	0.00	0.01	0.00
ILE	33 (+3)	0.01	0.01	0.01	0.00	0.01	0.00	0.00	0.00
ASP	47 (0)	-1.59	0.22	-1.48	0.07	-1.56	0.15	-1.50	0.11
ASP	48 (0)	-10.53	0.40	-13.90	0.46	-14.32	0.49	-14.60	0.61
GLY	49 (-2)	-0.12	0.01	-0.20	0.01	-0.24	0.06	-0.21	0.01
PRO	50 (-2/-3)	-0.06	0.00	-0.06	0.00	-0.11	0.03	-0.10	0.01
SER	51 (-3)	0.00	0.00	0.01	0.01	-0.04	0.09	-1.13	0.07
SER	52 (-3)	0.00	0.00	0.02	0.00	0.03	0.01	-0.72	0.11
TYR	53 (-3)	0.01	0.00	0.00	0.00	-0.04	0.04	-0.64	0.07

Table S5: A small section of the hydrogen bond occupancy of all substrates in all binding modes. The complete table is included in the supporting material as an additional spreadsheet.

donor	acceptor	conf7_1st	conf7_1st	conf7_3rd	conf11_1st	conf11_2nd	conf11_3rd	conf16_1st	conf16_2nd	conf16_3rd	average	standard_	subsite
0YB5-Side-O3	ASP48-Side-OD2	89.31%	90.37%	91.09%	91.96%	95.87%	92.08%	95.05%	90.71%	94.87%	92.37%	2.20%	0
TYR138-Main-N	0YB5-Side-O2N	94.58%	89.06%	93.69%	84.80%	90.77%	96.20%	94.19%	94.01%	92.97%	92.25%	3.31%	0
HIS195-Side-NE2	0YB5-Side-O4	87.63%	14.92%	47.15%	11.80%	85.87%	17.23%	61.03%	52.02%	89.21%	51.87%	30.01%	0
4YB4-Side-O6	ASP162-Side-OD2	22.70%	19.52%	13.00%	11.97%	5.37%	31.08%	9.26%	56.20%	18.98%	20.90%	14.44%	1
LYS164-Side-NZ	4YB3-Side-O3	11.16%	13.08%	6.62%	4.21%	8.33%	15.85%	4.28%	11.22%	14.62%	9.93%	4.06%	2
4YB4-Side-O6	ASP162-Side-OD1	3.86%	0.39%	2.46%	0.37%	3.01%	0.45%	30.31%	4.09%	9.41%	6.04%	8.98%	1
LYS164-Side-NZ	4YB4-Side-O2N	4.37%	3.96%	4.96%	2.27%	3.36%	7.76%	7.57%	8.03%	0.79%	4.79%	2.41%	1
ASN167-Side-ND2	4YB4-Side-O2N	1.56%	3.59%	16.26%	8.63%	2.92%	3.75%	4.49%	1.01%	0.33%	4.73%	4.68%	1
LYS164-Side-NZ	4YB4-Side-O6	3.77%	8.50%	0.80%	1.60%	2.28%	12.65%	2.54%	3.32%	5.59%	4.56%	3.60%	1
4YB3-Side-O3	TYR166-Side-OH	0.00%	0.19%	4.54%	9.37%	0.95%	0.09%	5.07%	1.94%	0.65%	2.53%	3.01%	2
4YB3-Side-O3	ASP162-Side-OD2	2.29%	0.35%	3.87%	2.67%	0.96%	0.02%	1.54%	4.11%	2.26%	2.01%	1.35%	2
LYS164-Side-NZ	4YB3-Side-O4	1.78%	0.79%	1.39%	1.28%	0.51%	1.78%	2.12%	2.39%	1.99%	1.56%	0.59%	2
0YB5-Side-O4	ASP48-Side-OD2	0.39%	1.27%	0.53%	9.43%	0.30%	0.77%	0.48%	0.31%	0.33%	1.53%	2.81%	0
TYR166-Side-OH	4YB4-Side-O6	0.56%	1.29%	1.09%	0.52%	0.36%	3.14%	2.13%	3.55%	0.66%	1.48%	1.12%	1

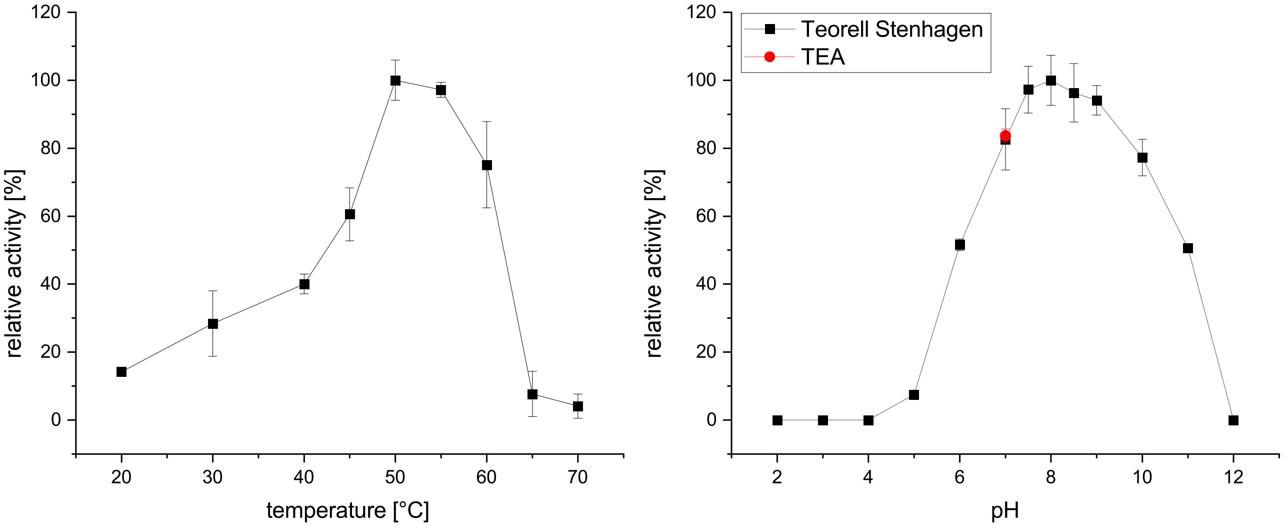

Figure S5: Temperature and pH optimum of AngCDA. The temperature optimum was determined at pH 7, while the pH optimum was determined at 37 °C. The relative activity corresponds to the highest conversion of A4 to A3D1, set to 100 % ($n = 3$).

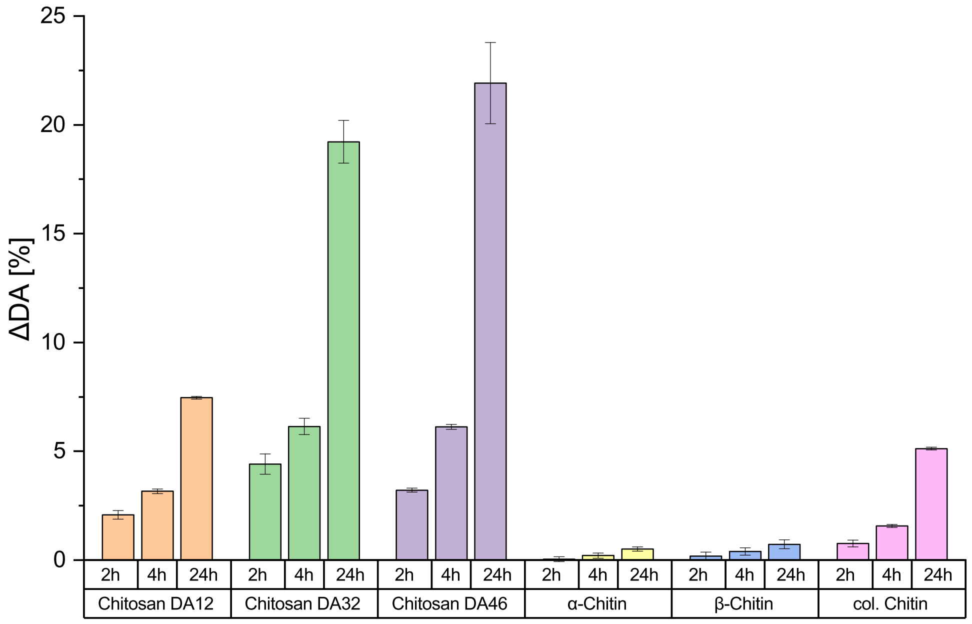

Figure S6: Activity on polymeric substrates including chitosans with different DA and chitinous substrates. The activity is given as the absolute change in DA after 2, 4 and 24 h ($n = 3$).
